# Temporal Variations in Cigarette Tobacco Bacterial Community Composition and Tobacco-Specific Nitrosamine Content Are Influenced by Brand and Storage Conditions

**DOI:** 10.3389/fmicb.2017.00358

**Published:** 2017-03-07

**Authors:** Jessica Chopyk, Suhana Chattopadhyay, Prachi Kulkarni, Eoghan M. Smyth, Lauren E. Hittle, Joseph N. Paulson, Mihai Pop, Stephanie S. Buehler, Pamela I. Clark, Emmanuel F. Mongodin, Amy R. Sapkota

**Affiliations:** ^1^Maryland Institute for Applied Environmental Health, School of Public Health, University of MarylandCollege Park, MD, USA; ^2^Institute for Genome Sciences and Department of Microbiology and Immunology, School of Medicine, University of MarylandBaltimore, MD, USA; ^3^Department of Biostatistics and Computational Biology, Dana-Farber Cancer InstituteBoston, MA, USA; ^4^Department of Biostatistics, Harvard T.H. Chan School of Public HealthBoston, MA, USA; ^5^Center for Bioinformatics and Computational Biology, University of MarylandCollege Park, MD, USA; ^6^Public Health Center for Tobacco Research, BattelleColumbus, OH, USA; ^7^Department of Behavioral and Community Health, School of Public Health, University of MarylandCollege Park, MD, USA

**Keywords:** cigarettes, tobacco, bacteria, bacterial community composition, microbiome, storage conditions, tobacco-specific nitrosamines, 16S rRNA gene

## Abstract

Tobacco products, specifically cigarettes, are home to microbial ecosystems that may play an important role in the generation of carcinogenic tobacco-specific nitrosamines (TSNAs), as well as the onset of multiple adverse human health effects associated with the use of these products. Therefore, we conducted time-series experiments with five commercially available brands of cigarettes that were either commercially mentholated, custom-mentholated, user-mentholated, or non-mentholated. To mimic user storage conditions, the cigarettes were incubated for 14 days under three different temperatures and relative humidities (i.e., pocket, refrigerator, and room). Overall, 360 samples were collected over the course of 2 weeks and total DNA was extracted, PCR amplified for the V3V4 hypervariable region of the 16S rRNA gene and sequenced using Illumina MiSeq. A subset of samples (*n* = 32) was also analyzed via liquid chromatography with tandem mass spectrometry for two TSNAs: *N*’-nitrosonornicotine (NNN) and 4-(methylnitrosamino)-1-(3-pyridyl)-1-butanone (NNK). Comparative analyses of the five tobacco brands revealed bacterial communities dominated by *Pseudomonas*, *Pantoea*, and *Bacillus*, with *Pseudomonas* relatively stable in abundance regardless of storage condition. In addition, core bacterial operational taxonomic units (OTUs) were identified in all samples and included *Bacillus pumilus, Rhizobium* sp., *Sphingomonas* sp., unknown *Enterobacteriaceae*, *Pantoea* sp., *Pseudomonas* sp., *Pseudomonas oryzihabitans*, and *P. putida*. Additional OTUs were identified that significantly changed in relative abundance between day 0 and day 14, influenced by brand and storage condition. In addition, small but statistically significant increases in NNN levels were observed in user- and commercially mentholated brands between day 0 and day 14 at pocket conditions. These data suggest that manufacturing and user manipulations, such as mentholation and storage conditions, may directly impact the microbiome of cigarette tobacco as well as the levels of carcinogens.

## Introduction

The tobacco microenvironment within cigarettes is home to complex mixtures of chemicals, metals, salts, trace pesticides, alkaloids, and commercial additives (e.g., menthol and sweeteners; Pauly and Paszkiewicz, 2011; Rodgman and Perfetti, 2013). In fact, over 5,000 components have been identified in tobacco and over 6,000 in tobacco smoke, many of which are carcinogenic toxins (Talhout et al., 2011; Rodgman and Perfetti, 2013). Among the potentially harmful constituents of tobacco are bacteria, fungi, and their microbially derived toxins (Pattee, 1969; Hasday et al., 1999; Pauly and Paszkiewicz, 2011). Multiple studies have shown that bacteria can not only survive the low moisture content of tobacco but also withstand the harsh smoking process (Eaton et al., 1995; Rooney et al., 2005; Pauly et al., 2008). Specifically, species of *Bacillus*, *Kurthia*, and *Mycobacterium* have been successfully recovered *in vitro* from cigarette filters, smoked filters, paper, and tobacco microparticulates (Eaton et al., 1995; Rooney et al., 2005; Pauly et al., 2008).

In addition, molecular techniques to assay the bacterial diversity of tobacco products have identified hundreds of bacterial species present in cured tobacco leaves (Di Giacomo et al., 2007; Huang et al., 2010; Su et al., 2011), cigarettes (Sapkota et al., 2010), and smokeless tobacco brands (Tyx et al., 2016). These comprise species from the families *Pseudomonadaceae, Staphylococcaceae*, *Lactobacillaceae*, *Enterobacteriaceae*, *Enterococcaceae*, *Aerococcaceae*, *Corynebacteriaceae*, among others, and include potential human and respiratory pathogens (Di Giacomo et al., 2007; Huang et al., 2010; Sapkota et al., 2010; Su et al., 2011). Furthermore, tobacco and tobacco smoke have been shown to harbor microbial derived toxins and secondary metabolites (Hasday et al., 1999). For instance, lipopolysaccharide, a potent inflammatory endotoxin of gram-negative bacteria, was identified as a bioactive component of cigarette smoke and a suggested cause of respiratory diseases among smokers (Hasday et al., 1999; Wendell and Stein, 2001; Larsson et al., 2004). These microbial components of the cigarette may be inhaled during use and deposited into the lung and oral cavity, where they may directly impact the health of the user.

Prior to packaging within the cigarette wrapper, tobacco is influenced heavily by bacteria. This occurs largely during the curing process, a necessary part of cigarette production, whereby tobacco leaves are dried generally by flue (e.g., Virginia tobacco), air (e.g., Burley tobacco), or sun (e.g., Oriental tobacco) to improve their color, flavor, and aroma (Leffingwell, 1999). During the curing stage, the amount of tobacco specific nitrosamines (TSNAs), carcinogens derived from the nitrosation of tobacco alkaloids, increases significantly (Wiernik et al., 1995). This is suggested to be, in part, due to certain nitrate and nitrite reducing bacterial species present on or in the tobacco leaves (Atawodi and Richter, 1996). High temperatures and relative humidities have been shown to be key factors that contribute to increasing levels of TSNAs throughout curing (Burton et al., 1989b; Law et al., 2016) and storage (Burton et al., 1989a; Shi et al., 2013) of tobacco. TSNA levels in smokeless tobacco brands have also been shown to be influenced by storage conditions, with high levels of TSNAs associated with storage for 4 weeks at room and high temperatures (>37°C), but not low temperature (4°C; Djordjevic et al., 1993). This may be due to changing bacterial diversity within these products.

Microbial populations are often dynamic and influenced by surrounding environmental conditions (Kinkel, 1997; Di Giacomo et al., 2007). For instance, changes in temperature, pH, and nutrient availability throughout the Toscano cigar tobacco fermentation cycle were shown to be associated with changes in the bacterial community composition of these products (Di Giacomo et al., 2007). In addition, storage conditions have also been found to influence microbial exposures of tobacco users. For example, cigarettes kept at high humidity have been characterized by increased levels of fungi (Larsson et al., 2008). However, to our knowledge there is no literature describing the longitudinal effects of varying storage conditions (e.g., temperature and relative humidity) on the bacterial diversity of cigarettes. Therefore, this study aimed to utilize high throughput 16S rRNA gene sequencing to investigate the bacterial community composition of five cigarette brands over 14 days at average room, refrigerator, and pocket conditions to identify potential trends in overall bacterial diversity and in specific operational taxonomic units (OTUs). In addition, a subset of samples was tested for levels of two TSNAs [*N*-nitrosonornicotine (NNN) and 4-(methylnitrosamino)-1-(3-pyridyl)-1-butanone (NNK)] at pocket and refrigerator conditions over time.

## Materials and Methods

### Sample Collection and Treatment

Five different cigarette brands (including three distinct lots per brand) were analyzed in this study. Camel Crush, regular, fresh (CC; R.J. Reynolds Tobacco Co., Winston-Salem, NC, USA) and Newport Menthols (NMB; Lorillard Tobacco Co., Greensboro, NC, USA) were purchased from tobacco stores in College Park, MD, USA. CC cigarettes, where the capsule within the filter was not crushed, were considered non-mentholated, while those where the capsule was crushed to release a menthol-containing solution into the cigarette filter were considered user-mentholated (CCM). Camel full flavor, hard pack, king (CK; R.J. Reynolds Tobacco Co., Winston-Salem, NC, USA) were provided by our collaborators at The Battelle Public Health Center for Tobacco Research (Columbus, OH, USA) along with a custom mentholated version (CKM) as described in MacGregor et al. (2014). To reflect normal user storage conditions cigarettes were subjected to 14 days of three different experimental storage conditions: pocket (25°C and 30% relative humidity), refrigerator (5°C and 18% relative humidity), and room (20°C and 50% relative humidity). Subsets of cigarettes (*n* = 6) were sampled from each brand for DNA extraction and 16S rRNA amplification prior to onset of the experimental condition (day 0), after 5 days, after 9 days, and after 14 days for each condition (Supplementary Table S1).

### DNA Extraction

Total DNA extraction was adapted from procedures previously published (Zupancic et al., 2012; Jackson et al., 2014). Cigarettes were dissected separately under sterile conditions and 0.2 g of tobacco was removed and aseptically placed in Lysing Matrix B tubes (MP Biomedicals, Solon, OH, USA). To achieve an effective enzymatic lysis, 1 ml of ice cold 1X molecular grade PBS buffer (Gibco by Life Technologies, Grand Island, NY, USA), 5 μl lysozyme from chicken egg white (10 mg/ml, Sigma-Aldrich, St. Louis, MO, USA), 5 μl lysostaphin from *Staphylococcus staphylolyticus* (5 mg/ml, Sigma-Aldrich, St. Louis, MO, USA) and 15 μl of mutanolysin from *Streptomyces globisporus* ATCC 21553 (1 mg/ml, Sigma-Aldrich, St. Louis, MO, USA) was added to the tubes containing cigarette tobacco and lysing matrix. Tubes were then incubated at 37°C for 30 min followed by the addition of a second enzymatic cocktail consisting of 10 μl Proteinase K (20 mg/ml, Invitrogen by Life Technologies, Grand Island, NY, USA) and 50 μl of SDS (10% w/v, BioRad). Incubation was repeated at 55°C for 45 min. Samples were then subjected to mechanical lysis via the FastPrep Instrument FP-24 (MP Biomedicals, Santa Ana, CA, USA) at 6.0 m/s for 40 s followed by centrifugation for 3 min at 10,000 rcf. Subsequent DNA was purified using the QIAmp DSP DNA mini kit 50, v2 (Qiagen, Valencia, CA, USA), according to the manufacturer’s protocol. Negative extraction controls were included to ensure that no exogenous DNA contaminated the samples during extraction. DNA quality control/quality assurance was performed using spectrophotometric measurements on a NanoDrop^TM^ (Thermo Scientific, Waltham, MA, USA), as well as gel electrophoresis.

### 16S rRNA Gene PCR Amplification and Sequencing

Using a dual-indexing strategy for multiplexed sequencing developed at the Institute for Genome Sciences and described in detail elsewhere (Fadrosh et al., 2014), the V3V4 hypervariable region of the 16S rRNA gene was PCR-amplified and sequenced on the Illumina MiSeq (Illumina, San Diego, CA, USA). PCR reactions were set-up in 96-well microtiter plates using the 319F (ACTCCTACGGGAGGCAGCAG) and 806R (GGACTACHVGGGTWTCTAAT) universal primers, each with a linker sequence required for Illumina MiSeq 300 bp paired-ends sequencing, and a 12-bp heterogeneity-spacer index sequence to minimize biases associated with low-diversity amplicons sequencing (Caporaso et al., 2012; Fadrosh et al., 2014). Reactions were performed with Phusion High-Fidelity DNA polymerase (Thermo Fisher, Waltham, MA, USA) and 2 ng of template DNA in a total reaction volume of 25 μl. In addition, due to the presence of PCR inhibitors, an additional 0.375 μl of bovine serum albumin (BSA; 20 mg/ml, Sigma) was added to the PCR reactions. Negative controls without DNA template were performed for each primer pair. A DNA Engine Tetrad 2 thermo cycler (Bio-Rad, USA) was used under the following cycling parameters: 30 s at 98°C, followed by 30 cycles of 10 s at 98°C, 15 s at 66°C, and 15 s at 72°C, with a final step of 10 min at 72°C. Successful amplification was confirmed using gel electrophoresis. This was followed by cleanup and normalization via the SequalPrep Normalization Plate kit (Invitrogen Inc., Carlsbad, CA, USA) with 25 ng of 16S PCR amplicons from each sample prior to pooling and 16S rRNA sequencing using the Illumina MiSeq (Illumina, San Diego, CA, USA) according to the manufacturer’s protocol.

### TSNA Analysis

Concentrations of two TSNAs (NNN and NNK) in the unused product were determined for a subset of cigarette samples (*n* = 32). The subset included two samples taken at day 0 and two samples taken at day 14 at pocket conditions for all five brands. In addition, two samples taken at day 0 and two samples taken at day 14 at refrigerator conditions for CK, CKM, and NMB were included. Samples were stored at -80°C until analysis. Prior to extraction, the tobacco and the outer wrapper (cut into small pieces) were removed, weighed separately, and then combined for analysis. Filters and the paper encasing them were removed and discarded.

Samples were extracted using a method adopted from those previously published for smokeless tobacco products (Stanfill et al., 2010; Lawler et al., 2013). Each sample was spiked with deuterated internal standards (NNN-d4 and NNK-d4) and extracted in 30 mL of ammonium acetate on a rotary shaker for 1 h at 250 rpm. Each extract was then filtered with a 0.45 mm syringe filter. Quality control samples, including matrix spikes, were prepared with each batch of samples using 3R4F cigarettes. Extracts were analyzed using liquid chromatography with tandem mass spectrometry (LC-MS/MS). The method detection limit based on average sample tobacco weights was 0.002 mg/g. Matrix spike recoveries averaged 113 ± 23% for NNN and 110 ± 9% for NNK.

### Sequence Quality Filtering

After screening 16S rRNA gene reads for low quality bases and short read lengths (Fadrosh et al., 2014) paired-end read pairs were then assembled using PANDAseq (Masella et al., 2012), de-multiplexed, trimmed of artificial barcodes and primers, and assessed for chimeras using UCHIME in *de-novo* mode implemented in Quantitative Insights Into Microbial Ecology (QIIME; release v. 1.9; Caporaso et al., 2010). The resulting quality trimmed sequences were then clustered *de-novo* into OTUs with the SILVA 16S database (Quast et al., 2012) in QIIME (Caporaso et al., 2010), with a minimum confidence threshold of 0.97 for the taxonomic assignments. All sequences taxonomically assigned to chloroplasts were removed. To account for uneven sampling depth and to ensure less biases than the standard approach (total sum normalization), data were normalized with metagenomeSeq’s cumulative sum scaling when appropriate (Paulson et al., 2013b).

### Data Analysis

Taxonomic assignments of genera were obtained through QIIME (Caporaso et al., 2010). After removing genera whose maximum relative abundance was less than 1%, a heatmap was created and visualized with R version 3.2.2 and vegan heatplus (Ploner, 2012). The core tobacco bacterial microbiome was defined as OTUs present at a minimum fraction of 100% in all tested products with QIIME’s compute_core_microbiome.py script (Caporaso et al., 2010) and visualized with Cytoscape (Shannon et al., 2003).

Beta diversity for all brands at all times and conditions was calculated using the Bray–Curtis dissimilarity and compared using Analysis of similarities (ANOSIM) on normalized data (999 permutations) through the R packages: biomformat (McMurdie and Paulson, 2015), vegan (Oksanen et al., 2007), ggplot2 (Wickham, 2009), phyloseq (McMurdie and Holmes, 2013). Beta diversity was also calculated as described above for samples separated by brand.

Diversity was estimated for samples pooled by brand, time point, and condition using the Shannon Index (Shannon et al., 2003) through the R packages: Bioconductor (Huber et al., 2015), metagenomeSeq (Paulson et al., 2013a), vegan (Oksanen et al., 2007), phyloseq (McMurdie and Holmes, 2013), and fossil (Vavrek, 2011). Significance was assessed through Tukey’s test at *p* < 0.05. To account for uneven sampling depth, diversity was measured with and data rarefied to a minimum sampling depth.

Determination of statistically significant differences (*p*-value < 0.001) in OTU abundance was performed using DESeq2 (Love et al., 2013, 2014) to compare the NMB brand between day 0 and day 14 at room, pocket, and refrigerator conditions. The significant OTUs (*p* < 0.001) were visualized with R version 3.2.2 and R packages ggplot2 (Wickham, 2009), vegan (Oksanen et al., 2007), and phyloseq (McMurdie and Holmes, 2013). This was repeated for the remaining brands (CC, CCM, CK, CKM), as well as, by product lot.

## Results

### Sequencing

DNA extraction and sequencing was performed on 360 cigarette samples (Supplementary Table S1), with a total of 2,172,847 sequences and an average sequence per sample of 6,262 (±3,433 SD). A total of 1,985 different bacterial OTUs (97% identity) were identified at an average of 185 OTUs per sample (±46 SD).

### Taxonomic Analysis of All Cigarette Brands

**FIGURE 1:**
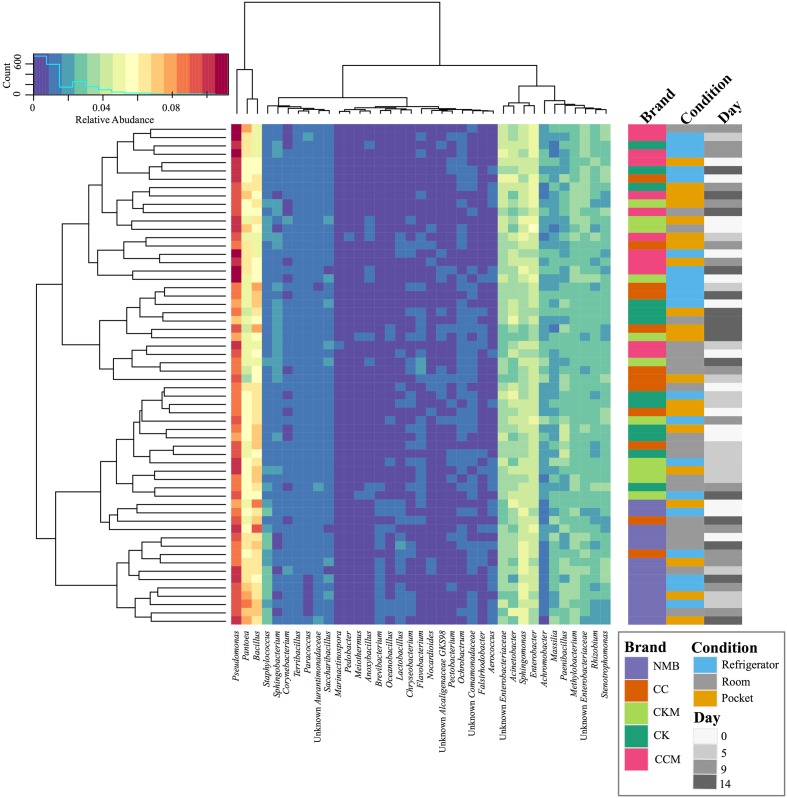
**Bacterial community composition of cigarette products over time and differing storage conditions**. Heat map showing the relative abundances of the most dominant bacterial genera identified (>1%) in cigarette products pooled by brand (CK, CKM, CC, CCM, and NMB), time point (day 0, day 5, day 9, and day 14), and experimental storage condition (room, pocket, refrigerator) and denoted by colored rectangles. Clustering using Manhattan distance of the pooled samples represented by the dendrograms.

After samples were pooled by brand (CC, CCM, CK, CKM, and NMB), time point (day 0, day 5, day 0, and day 14), and condition (pocket, room, and refrigerator), *Pseudomonas* had the highest relative abundance in all instances, ranging from 7.05 to 11.24%. This was followed by either *Pantoea* (3.58–8.44%) or *Bacillus* (4.58–9.38%) (**Figure 1**). These three encompassed the furthest clade to the left of the cladogram (**Figure 1**). The second most abundant clade of bacterial genera consisted of *Acinetobacter* (2.16–4.84%), *Enterobacter* (3.09–5.27%), Unknown *Enterobacteriaceae* (2.53–4.76%), and *Sphingomonas* (2.97–5.13%) (**Figure 1**).

**FIGURE 2:**
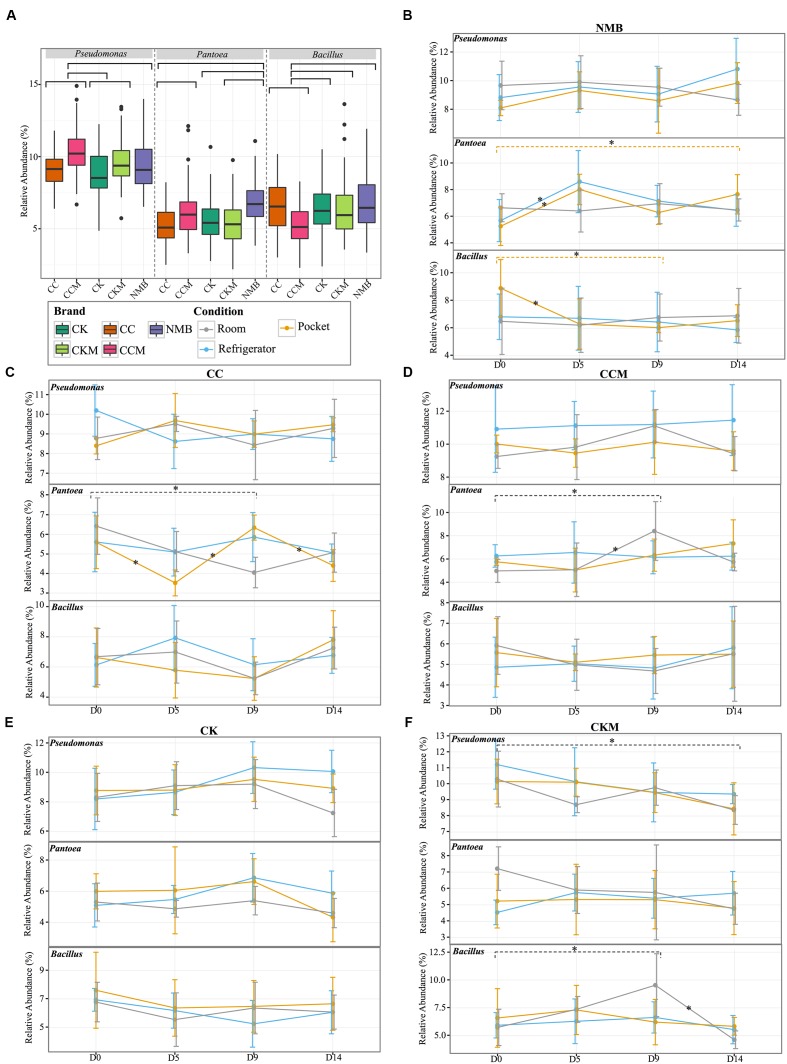
**Comparison of the relative abundance of the most dominant genera**. **(A)** Boxplot of the relative abundance of the most dominant genera (*Pseudomonas*, *Pantoea*, *Bacillus*) in each brand (CC, CCM, CK, CKM, NMB). Brands are colored as follows: CC (dark orange), CCM (pink), NMB (purple), CKM (light green), CK (dark green). Line graphs with standard deviations of relative abundances of the same genera within brand **(B)** NMB, **(C)** CC, **(D)** CCM, **(E)** CK, **(F)** CKM over time and experimental storage condition. Experimental storage condition denoted by color as follows: room (gray), pocket (orange), and refrigerator (light blue). Asterix on lines and dashed brackets represent significant changes between time points. Significance determined by an alpha level of 0.05.

When samples were pooled by brand (**Figure 2A**) *Pseudomonas* was significantly (*p* < 0.05) higher in relative abundance in CCM compared to CC, CK, and NMB. CCM also had a significantly higher relative abundance of *Pantoea* than CC and a significantly lower relative abundance of *Bacillus* than CC, CK, CKM, and NMB. Furthermore, CKM had significantly higher relative abundance of *Pseudomonas* than CK. NMB had a significantly higher relative abundance of *Pantoea* than CC, CK, CKM.

Within brand condition was also a prominent factor impacting the temporal dynamics of the most abundant genera (**Figures 2B–F**). Experimental condition seemed to have little significant effect on the relative abundance of *Pseudomonas* over time. In fact, *Pseudomonas* only significantly changed in one brand, CKM, in which it decreased between day 0 and day 14 at room conditions (**Figure 2F**). The relative abundance of *Bacillus* was only affected by condition in NMB at pocket conditions and CKM at room conditions. For CKM there was a significant increase in *Bacillus* between day 0 and day 9 at room conditions, followed by a decrease between day 9 and day 14 (**Figure 2F**). For NMB, *Bacillus* decreased in relative abundance between day 0 and day 5 and then stayed relatively unchanged for the remainder of the study (**Figure 2B**).

The relative abundance of *Pantoea* appeared to be more affected by condition, whereas changes in the relative abundance occurred in CC at pocket and room conditions (**Figure 2C**), in CCM at room conditions (**Figure 2D**), and in NMB at pocket and refrigerator conditions (**Figure 2B**). Specifically, for NMB there was a significant increase in the relative abundance of *Pantoea* between day 0 and day 14 and between day 0 and day 5 at pocket conditions, with an oscillation downward at day 9 (**Figure 2B**). In addition, there was a significant increase in the relative abundance of *Pantoea* between day 0 and day 5 at refrigerator conditions for the same brand.

For CC, the relative abundance of *Pantoea* significantly fluctuated between day 0 and day 5, day 5 and day 9, and day 9 and day 14 at pocket conditions. There was also a significant decrease in *Pantoea* between day 0 and day 9 for CC at room conditions (**Figure 2C**). This is in contrast to CCM in which there was a significant increase in *Pantoea* between those same times at the same condition (**Figure 2D**).

**FIGURE 3:**
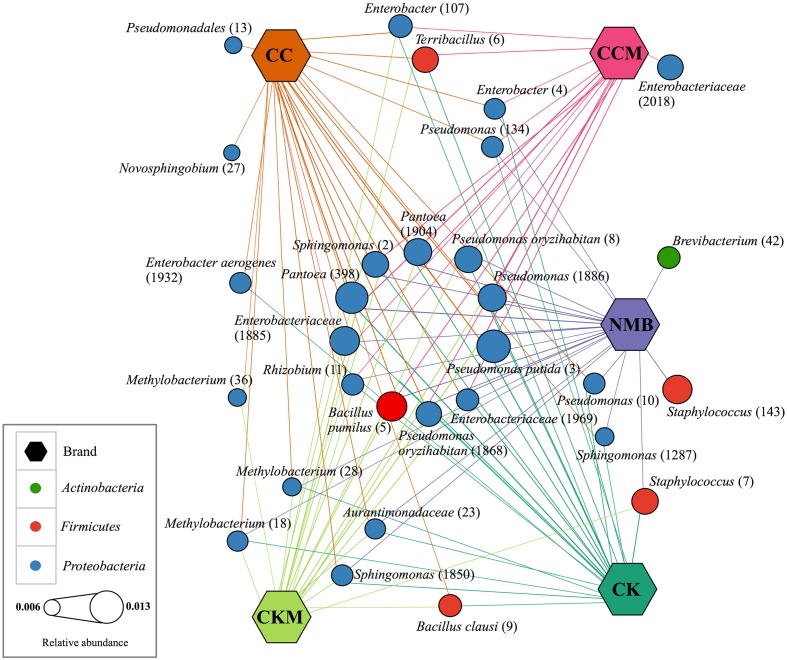
**Network of core bacterial operational taxonomic units (OTUs) in each brand**. Cytoscape network visualizing the OTUs (circles) that occur in 100% of samples (hexagon) pooled by brand and labeled with taxonomic ID and assigned OTU number in parantheses. Brands are colored as follows: CC (dark orange), CCM (pink), NMB (purple), CKM (light green), CK (dark green). Size of OTU nodes represent relative abundance and are colored by phylum: *Actinobacteria* (green), *Firmicutes* (red), and *Proteobacteria* (blue). Inner circle of nodes represent eleven core bacterial OTUs that occur in all samples, regardless of brand, time point or condition.

The core microbiome, defined for each brand, comprised 26 bacterial OTUs for CC, 24 for CK, 22 for NMB, 20 for CKM, and 16 for CCM (**Figure 3**). A comparative analysis of these bacterial OTUs revealed that 11 OTUs were shared among all samples regardless of brand, time, and experimental condition at relative abundances between 1.26% (*Pseudomonas putida*, OTU #3) and 0.83% (*Rhizobium* sp., OTU #11). These included: *B. pumilus* (OTU #5), *Rhizobium* sp. (OTU #11), *Sphingomonas* sp. (OTU #2), unknown *Enterobacteriaceae* (OTU #1969 and #1885), *Pantoea* sp. (OTU #398 and #1904), *Pseudomonas* sp. (OTU #1886), *Pseudomonas* oryzihabitans (OTU #1868 and #8), and *P. putida* (OTU #3) (**Figure 3**).

Two OTUs were unique to the core of NMB, *Brevibacterium* sp. (OTU # 42) and *Staphylococcus* sp. (OTU # 143). Similarly two OTUs were unique to the core of CC, *Novosphingobium* sp. (OTU # 27) and unknown *Pseudomonadales* (OTU # 13). Only one OTU was unique to CCM, unknown *Enterobacteriaceae* (OTU # 2018), and there were no OTUs in the core microbiome unique to CKM and CK. The largest degree of overlap was between NMB, CC, CK, and CKM, which had an additional four OTUs in common amongst their core microbiomes: *Sphingomonas* sp. (OTU # 1850), *Methylobacterium* (OTU # 28 and # 18), and unknown *Aurantimonadaceae* (OTU #23). The two non-mentholated brands (CC and CK) both had *Enterobacter aerogenes* (OTU # 1932) amongst their core microbiomes. *Enterobacter* sp. (OTU # 4) and *Pseudomonas* sp. (OTU # 134) were a part of the core in all brands except the custom mentholated Camel Kings (CKM). The custom mentholated and non-mentholated Camel Kings (CKM and CK) along with the NMB each had *Staphylococcus* sp. (OTU # 7). *Terribacillus* sp. (OTU # 6) and *Enterobacter* sp. (OTU # 107) were a part of the core microbiomes of all brands except commercially mentholated NMB. CKM, CK, and CC all had *B. clausi* (OTU # 9), whereas *Pseudomonas* (OTU # 10) and *Sphingomonas* (OTU # 1287) were in the core microbiomes of NMB, CK, and CC. In addition, *Methylobacterium* (OTU # 36) was present in CC and CKM.

### Beta and Alpha Diversity of All Brands

**FIGURE 4:**
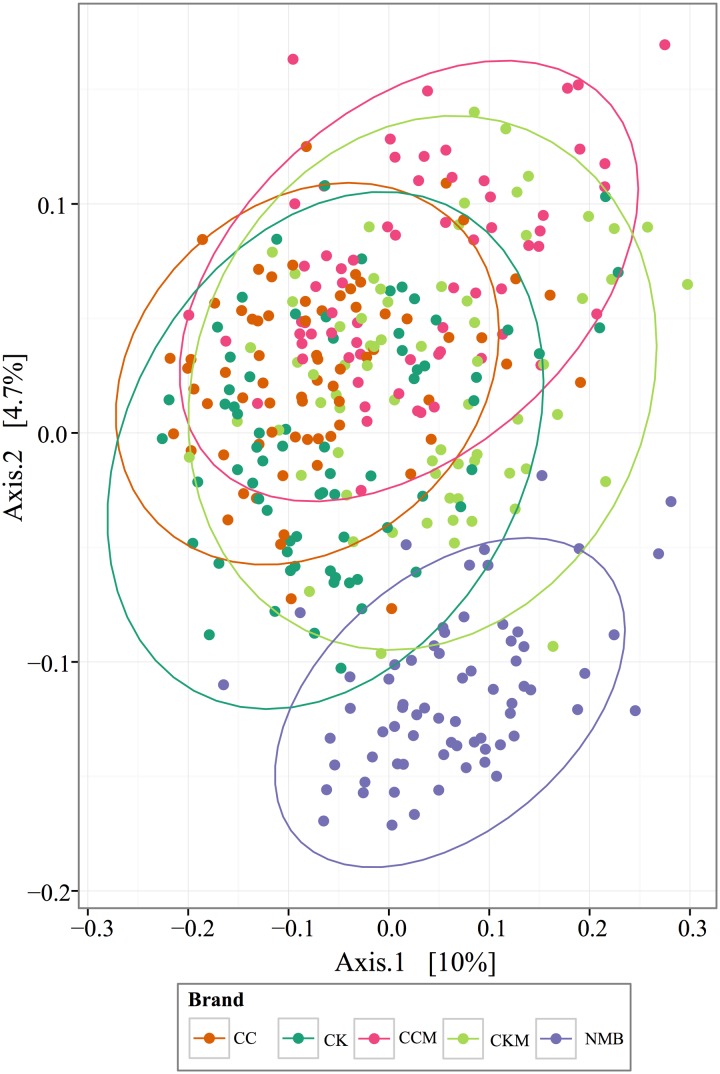
**PCoA analysis plots of Bray-Curtis computed distances between cigarette products**. Colored by brand and tested with ANOSIM (*R* = 0.28, *p* = 0.001). Ellipses are drawn at 95% confidence intervals for product brand.

PCoA plots of the Bray-Curtis computed beta diversity for all brands revealed the largest significant clustering by brand (*R* = 0.25, *p* = 0.001) followed by lot (*R* = 0.21, *p* = 0.001) (**Figure 4** and Supplementary Figure S1), with NMB observed clustering away from the other brands. There was no significant clustering by time point or condition (Supplementary Figure S1). When separated into distinct brands, each had minimum clustering by time point and lot (Supplementary Figure S2), particularly for CK (*R* = 0.1762, *p* = 0.001), CKM (*R* = 0.1703, *p* = 0.001), and NMB (*R* = 0.198, *p* = 0.001) lots.

All brands appeared to have fluctuating bacterial diversity, assessed through Shannon indices, during the length of the experiment (day 0, day 5, day 9, and day 14; Supplementary Figure S3). However, the only significant change in Shannon indices was between day 0 and day 9 in NMB at pocket conditions in which diversity increased (*p* < 0.05) (Supplementary Figure S3).

### Comparative Analysis of OTUs by Condition between Day 0 and Day 14

**FIGURE 5:**
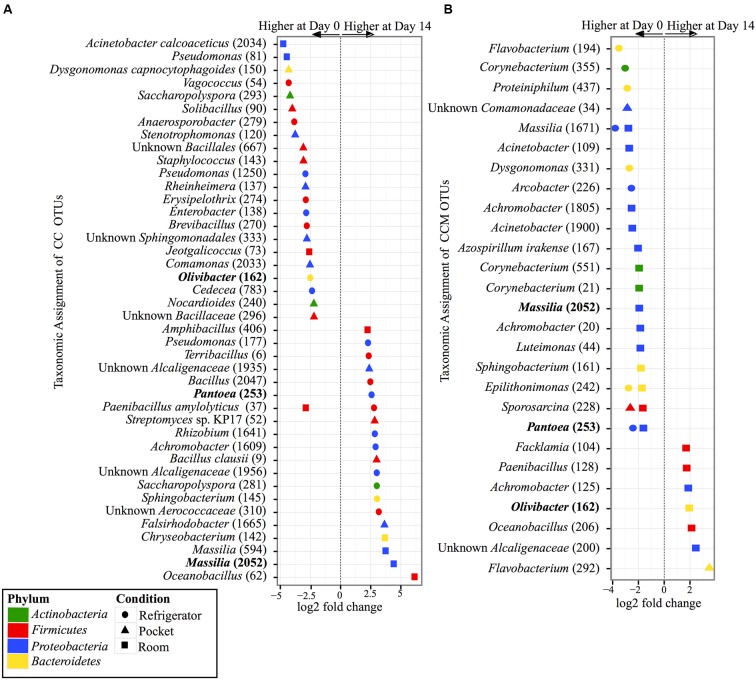
**Overview of relative abundances of bacterial OTUs that were statistically significantly different (*p*-value < 0.001) between day 0 and day 14 for refrigerator (circle), room (square), and pocket (triangle) conditions for (A)** non-mentholated Camel Crush (CC) and **(B)** mentholated Camel Crush (CCM). OTUs are colored by Phylum and shaped by experimental condition. A positive log2-fold change value denotes an OTU that is significantly higher at day 14, while a negative log2-fold change indicates an OTU that is significantly higher at day 0. The dotted line and arrows highlight the conversion in log2-fold change from negative to positive values. Bolded text refers to OTUs that occur in both **(A**,**B)**.

Within the experimental conditions tested, non-mentholated CC had the greatest amount of OTUs (19 OTUs) that were significantly different in relative abundance between day 0 and day 14 at refrigerator conditions (**Figure 5A**). Of these, 61% (11 OTUs) were at higher relative abundance at day 14 and the rest (8 OTUs) were at higher relative abundance at day 0. This was followed by pocket conditions, which had 15 OTUs significantly different between day 0 and day 14, with 73% (11 OTUs) at higher relative abundance at day 0. Room conditions had the least amount of significantly different OTUs (nine OTUs) between time points, of which 55% (five OTUs) were at higher relative abundance at day 14.

In contrast to its non-mentholated counterpart, CCM had the greatest number of OTUs (20 OTUs) that were significantly different between day 0 and day 14 at room conditions (**Figure 5B**), with 70% (14 OTUs) at higher abundance at day 0 compared to day 14. Refrigerator conditions had the second largest amount of OTUs (eight OTUs) that were significantly different between day 0 and day 14 for CCM, all of which had higher relative abundance at day 0. At pocket conditions there were only three OTUs that were significantly different between time points. Two were at higher abundance at day 0 and one was at a higher abundance at day 14.

**FIGURE 6:**
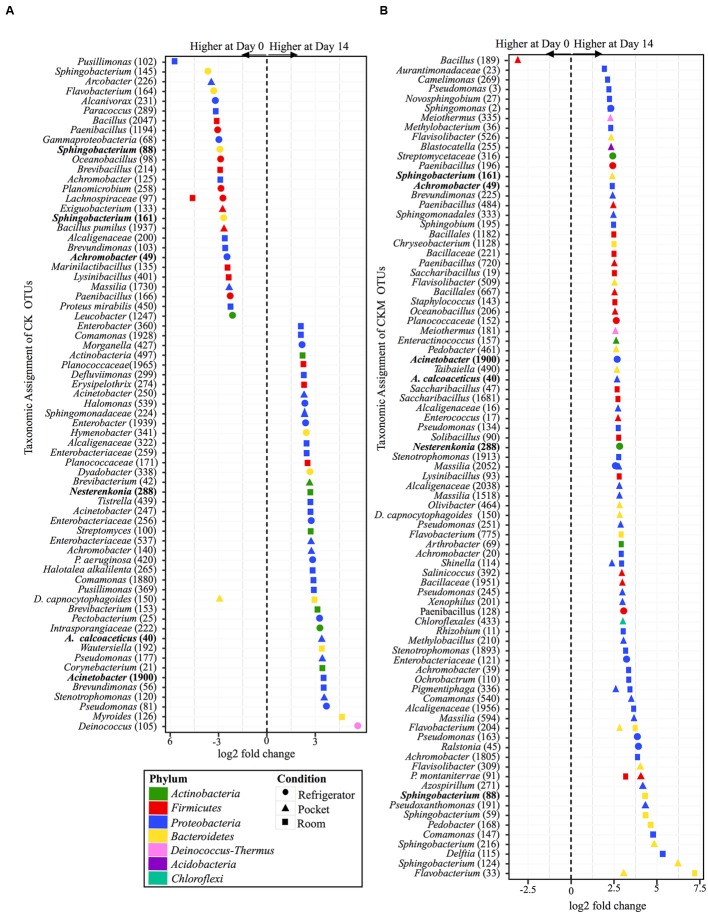
**Overview of relative abundances of bacterial OTUs that were statistically significantly different (*p*-value < 0.001) between day 0 and day 14 for refrigerator (circle), room temperature (square), and pocket (triangle) conditions for (A)** non-mentholated Camel Kings (CK) and **(B)** mentholated Camel Kings (CKM). OTUs are colored by Phylum and shaped by experimental condition. A positive log2-fold change value denotes an OTU that is significantly higher at day 14, while a negative log2-fold change indicates an OTU that is significantly higher at day 0. The dotted line and arrows highlight the conversion in log2-fold change from negative to positive values. Bolded text refers to OTUs that occur in both **(A**,**B)**.

Similar to CCM, non-mentholated Camel Kings (CK) had the largest amount of OTUs at significantly different relative abundances (34 OTUs) between day 0 and day 14 at room conditions (**Figure 6A**). However, unlike CCM, 67% of the OTUs (23 OTUs) were at higher relative abundance at day 14. The second condition that produced the most OTUs with significantly different relative abundances (24 OTUs) between time points was refrigerator conditions; 54% of OTUs (13 OTUs) at higher relative abundance at day 0. Pocket conditions had the smallest amount of OTUs at significantly different relative abundances (14 OTUs). Of these, 57% (eight OTUs) were at higher relative abundance at day 14.

Custom-mentholated Camel Kings (CKM) at pocket conditions had the most OTUs (43 OTUs) that were significantly different in relative abundance between day 0 and day 14 (**Figure 6B**). However, only one of these OTUs was at higher relative abundance at day 0, *Bacillus* (189). The remaining 98% (42 OTUs) were at higher relative abundance at day 14. Room conditions had 38 OTUs at significantly different relative abundance between time points, all at higher relative abundance at day 14. Finally, refrigerator conditions had the least number of OTUs (11 OTUs) that were significantly different in relative abundance between day 0 and day 14, all of which were higher at day 14.

**FIGURE 7:**
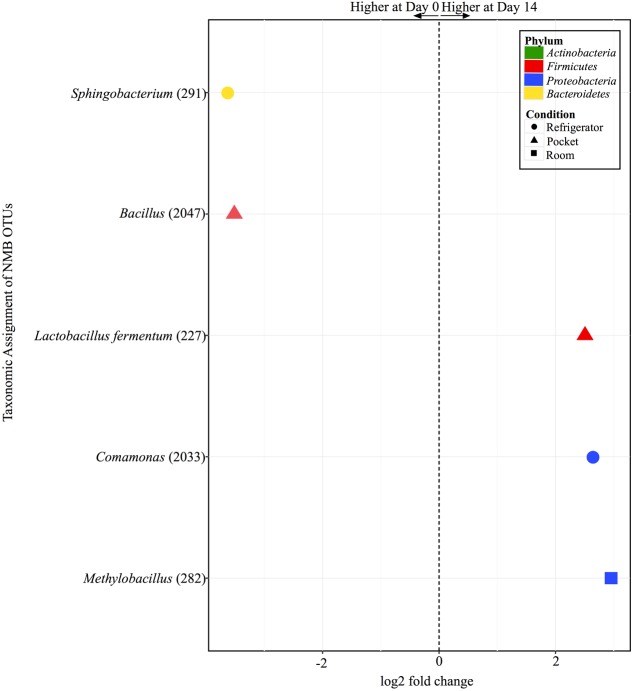
**Overview of relative abundances of bacterial OTUs that were statistically significantly different (*p*-value < 0.001) between day 0 and day 14 for refrigerator (circle), room temperature (square), and pocket (triangle) conditions for Newport Menthols (NMB)**. OTUs are colored by Phylum and shaped by experimental condition. A positive log2-fold change value denotes an OTU that is significantly higher at day 14, while a negative log2-fold change indicates an OTU that is significantly higher at day 0. The dotted line and arrows highlight the conversion in log2-fold change from negative to positive values.

There were only five OTUs at statistically significantly different (*p* < 0.001) relative abundances between day 0 and day 14 among the different conditions for NMB (**Figure 7**). Pocket and refrigerator conditions each had two OTUs that were at significantly different relative abundance between time points. In both conditions one of the OTUs was at higher relative abundance at day 0 and one higher at day 14. Room conditions had only one OTU, significantly higher at day 14.

Operational taxonomic units that are significantly different in relative abundance between day 0 and day 14 in both CC and CCM and in both CK and CKM are described in detail in the Supplementary Material, in addition to a comparison of the product lots (Supplementary Figures S4–S6).

### Analysis of TSNA Content

**FIGURE 8:**
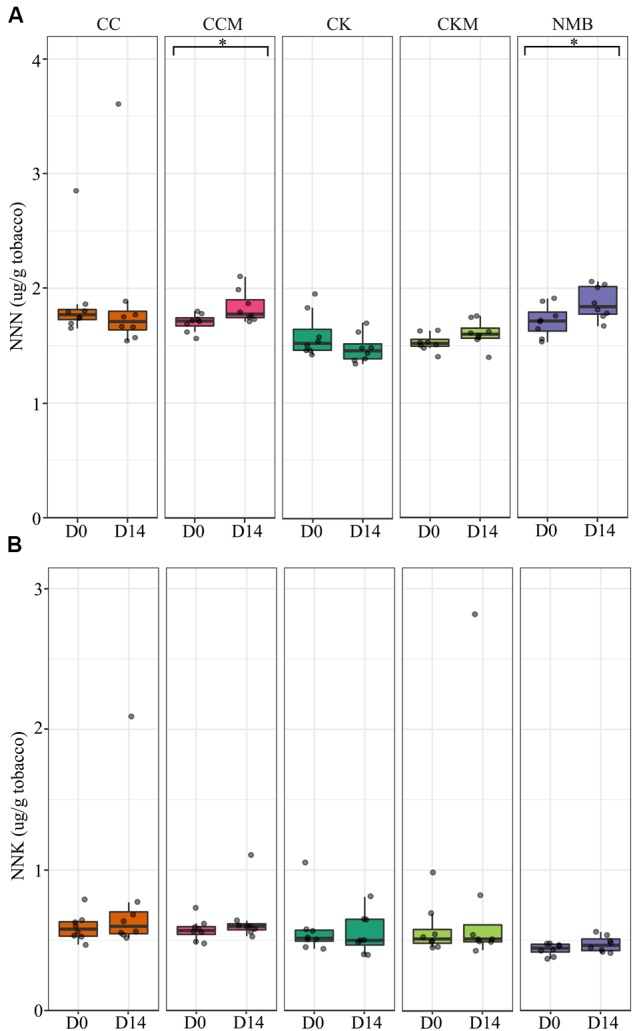
**Tobacco-specific nitrosamine levels over time at pocket conditions**. Comparison of **(A)** *N*-nitrosonornicotine (NNN) and **(B)** Nicotine-derived nitrosamine ketone (NNK) levels in all brands at day 0 (D0) and day 14 (D14) at pocket conditions. Significance at *p* < 0.05 shown by brackets at the top of the plot.

*N*-nitrosonornicotine levels were significantly higher *(p* < 0.05) at pocket conditions from day 0 to day 14 for NMB and CCM (**Figure 8A**). NNK levels increased as well from day 0 to day 14 for NMB and CCM; however, these results were not statistically significant (**Figure 8B**). Only CKM, CK, and NMB were tested for these TSNAs at refrigerator conditions (Supplementary Figure S7). NNN tended to increase in all brands from day 0 to day 14, while NNK levels tended to decrease in all brands over the same time. However, these differences were also not statistically significant.

## Discussion

Fresh tobacco leaves are colonized by a variety of microorganisms (Larsson et al., 2008) that can be altered by tobacco-processing methods following harvest, such as curing and fermentation (Di Giacomo et al., 2007; Zhao et al., 2007). However, the effect of storage conditions on the bacterial constituents of tobacco after packaging within a cigarette was previously unknown. Here, we showed that the dominant bacterial genera (**Figure 2**), specific OTUs (**Figures 5**–**7**), and the concentration of TSNAs (**Figure 8**) are related to the cigarette brand and the storage condition.

*Pseudomonas* was the most abundant bacterial genera detected in all brands, time points, and conditions (**Figure 1**). This corroborates with previous findings suggesting that *Pseudomonas* was a dominant bacterial genera on aged and unaged flue-cured tobacco leaves (Huang et al., 2010; Su et al., 2011). In addition, storage condition seemed to have little significant effect on the relative abundance of *Pseudomonas* over time, whereas *Pantoea* appeared more sensitive to storage condition (**Figure 2**). This may be indicative of differing colonization strategizes between the two genera (Sabaratnam and Beattie, 2003).

In addition, OTUs of *Pseudomonas* and *Pantoea* were both defined as members of the “core microbiome” of all products (**Figure 3**). *Pseudomonas* and *Pantoea* are gram-negative, which may contribute to the high levels of lipopolysaccharide found in cigarette tobacco and smoke (Hasday et al., 1999). Both genera also contain species that are associated with disease in humans (Hatchette et al., 2000; Musher, 2001; Dutkiewicz et al., 2016). These include *P. putida* and *P. oryzihabitans*, which are generally considered opportunist pathogens (Yang et al., 1996), particularly *P. oryzihabitans* which has been linked to bacteremia, peritonitis, and pneumonia (Lin et al., 1997).

Many of the members of the core microbiome were also present in the core microbiome defined for air-cured burley tobacco including *Pantoea*, *Pseudomonas*, *Sphingomonas*, and *Bacillus* (Law et al., 2016). Despite this agreement in core members between products, our results showed there was some divergence in bacterial community composition between brands of cigarettes. For instance, NMB had a larger degree of the genera *Staphylococcus* (**Figures 1**, **2**). A well known pathogenic species of *Staphylococcus*, *S. aureus*, has been found to have higher nasal carriage rates in smokers (Durmaz et al., 2001; Choi et al., 2006). This bacteria has also been shown to increase biofilm formation and host cell adherence in the presence of cigarette smoke (Kulkarni et al., 2012).

In addition, levels of the TSNA NNN were found in this study to increase significantly between day 0 an day 14 at pocket conditions for NMB and CCM, a potential public health concern given that carcinogen exposure has been found to correlate with the levels of TSNAs in smokeless tobacco products. Specifically, it has been reported that NNK and NNN nitrosamine biomarkers in the urine of smokeless tobacco users increased 32 and 12%, respectively, for every one-unit (μg/g wet wt) increase in NNK and NNN levels within their smokeless tobacco products (Hatsukami et al., 2015). In tobacco, bacteria have been identified that are capable of reducing nitrate to nitrite for the formation of TSNAs, including species of *Bacillus*, *Staphylococcus*, and *Corynebacterium* (Di Giacomo et al., 2007; Fisher et al., 2012). However, we are unable to determine with these data whether the OTUs present in our samples have such capabilities or were responsible for the observed increases in TSNAs levels. In addition, the type of tobacco and the subsequent nitrate availability, may factor into the ecology of TSNA production. For example, flue-cured and sun-cured tobaccos have been reported to have lower nitrate levels than air-cured (Sophia et al., 1989; Centers for Disease Control, and Prevention, 2010). The different tobacco varietals are also blended in various assortments by commercial manufacturers, often with additives (e.g., menthol), thereby resulting in varied nitrate levels and potentially different arrangements of the microbial community compositions (Ding et al., 2008). Keeping these variables in mind, more work is necessary to explore the potential connections between nitrate reducers in tobacco, such as *Lactobacillus fermentum* (**Figure 7**; Xu and Verstraete, 2001), and increasing levels of TSNAs.

Several studies have suggested that smoking tobacco products can alter the microbiome of the user by disrupting commensal bacterial populations, enabling the invasion of pathogens in an otherwise occupied niche (Bizzarro et al., 2013; Thomas et al., 2014; Wu et al., 2016). However, the relationship between the microbiome of the products and the user is just beginning to be explored. Here, we present evidence that cigarette tobacco is a dynamic microenvironment, with significant changes in members of the dominant bacterial genera (**Figure 2**), specific OTUs (**Figures 5**–**7**), and the concentration of TSNAs (**Figure 8**) dependent on brand, storage conditions, and time. In addition, bacterial genera present at high abundance in these products are also those common to respiratory infections among smokers (Hatchette et al., 2000; Durmaz et al., 2001; Musher, 2001; Choi et al., 2006). Although the capabilities of bacterial growth in cigarette filters post-smoking have been demonstrated (Eaton et al., 1995), our data currently cannot ascertain whether the bacteria found in the cigarette tobacco are capable of colonizing the oral and/or lung cavities of the user. Despite this uncertainty, their potential role in TSNA and toxin production makes them a potentially appropriate target for intervention.

## Availability of Data

Data concerning the samples included in this study are deposited in the NCBI BioProject database^1^ under BioProject accession numbers PRJNA311925 and PRJNA377117.

## Author Contributions

JC performed bioinformatic analyses, wrote and edited the manuscript. SC, PK, and LH performed the time series experiments. ES, JP, and MP participated in data analyses, interpretation of the results and manuscript editing. SB led the tobacco-specific nitrosamine analyses, interpreted results and wrote portions of the manuscript. PC contributed to the study design, data interpretation and manuscript review. EM and AS conceived the study design and protocol development, and contributed to data analysis, interpretation, and manuscript preparation. All authors read and approved the final manuscript.

## Conflict of Interest Statement

The authors declare that the research was conducted in the absence of any commercial or financial relationships that could be construed as a potential conflict of interest.

## Footnotes

1 https://www.ncbi.nlm.nih.gov/bioproject/
