# Supplementary material for: Temporal Variations in Cigarette Tobacco Bacterial Community Composition and Tobacco-Specific Nitrosamine Content Are Influenced by Brand and Storage Conditions
Source: Front Microbiol. 2017 Mar 7;8:358. doi: 10.3389/fmicb.2017.00358 (PMC5339245; doi:10.3389/fmicb.2017.00358)
Supplement: Supplementary file 1 [file Table_1.DOCX]

**Supplemental text:**

**Comparison of OTUs significantly different in relative abundance between day 0 and day 14 in CC and CCM**

Interestingly, there were some OTUs that were shared between CC and CCM.

For instance, *Massilia* (OTU #2052) was at higher relative abundance at day 0 in CCM at room temperature, however the same OTU was at higher relative abundance at day 14 in CC at the same condition. Additionally, *Olivibacter* (OTU #162) was at a higher relative abundance at day 14 at room temperature in CCM and higher relative abundance at day 0 at refrigerator conditions in CC. *Pantoea* (OTU #253) was at lower relative abundance at day 14 at both room temperature and refrigerator conditions in CCM, but was at higher relative abundance at day 14 for CC at refrigerator conditions.

**Comparison of OTUs significantly different in relative abundance between day 0 and day 14 in CK and CKM**

Several OTUs significantly different in relative abundance between day 0 and day 14 were shared between CK and CKM including, *Nesterenkonia* (OTU #288) and *Acinetobacter* (OTU #1900), which were at a higher relative abundance at day 14 at room temperature and refrigerator conditions, respectively. *Acinetobacter calcoaceticus* (OTU #40) was higher in both brands at day 14 at pocket conditions. *Achromobacter* (OTU #49) was higher at day 14 for CKM at room temperature, but higher at day 0 for CK at refrigerator conditions.

*Sphingobacterium* (OTU #88*)* was at higher relative abundance at day 0 in CK at refrigerator conditions, but was higher at day 14 in CKM at room temperature. *Sphingobacterium* (OTU #161*)* was also at higher relative abundance at day 0 in CK at refrigerator conditions, but was higher at day 14 in CKM at pocket conditions.

**Comparative analysis of significantly different OTUs by lot**

Because there was clustering by lot for CK, CKM, and NMB (Figure S2), we determined the OTUs that were statistically significantly different between lots, regardless of condition or time point. For NMB, lot 4K01 clustered away from lot 4C03 and lot 4C17, therefore 4K01 was compared with 4C03 and 4C17. There were 11 OTUs at statistically significantly different (*p* < 0.001) relative abundances between 4K01 and 4C03 (Figure S5). Of these 11 OTUs, 5 from the phylum Actinobacteria had a higher relative abundance in 4C03 compared to 4K01 including *Ochrobactrum* sp. (OTU #110), *Marinactinospora* sp. (OTU #113), *Brevibacterium* sp. (OTU #153), *Saccharopolyspora* sp. (OTU #281), and *Enteractinococcus* sp. (OTU #157). The remaining 6 from the phylum Firmicutes had a higher relative abundance in 4K01: *Caldalkalibacillus* sp. (OTU #261), *Kurthia* sp. (OTU #180), *Lactobacillus mucosae* (OTU #234), *Lactobacillus* sp. AB5262 (OTU #118), *Lactobacillus fermentum* (OTU #227), and *Pediococcus* sp. (OTU #50). These OTUs were also significantly higher in relative abundance in 4K01 compared to 4C17 along with *Pantoea* sp. (OTU #725 and #229), *Bacillus coagulans* (OTU #99), *Geobacillus* sp. (OTU #119), *Lactobacillus* sp. (OTU #301), *Paenibacillus* sp. (OTU #196), and *Streptomyces* sp. KP17 (OTU #52).

For CK and CKM, lot A4 clustered away from lots Il and L1, therefore A4 was compared with Il and L1. There were 21 OTUs at statistically significantly different (*p* < 0.001) relative abundances between A4 and L1 for CK, of which 8 were at higher abundance in L1 and 13 were at higher abundance in A4 (Figure S6). Six of the OTUs of higher abundance in A4 were also at higher abundance when comparing lot A4 with lot Il, *Tistrella* (OTU #439), *Azospirillum* *irakense* (OTU #167), *Pseudomonas* (OTU #177), *Pectobacterium* (OTU #25), *Wautersiella* (OTU #192), *Alcaligenes* (OTU #117), *Pediococcus* (OTU #50), and *Rheinheimera* (OTU #137)*.* There were 33 OTUs at statistically significantly different (*p* < 0.001) relative abundances between lots A4 and L1 for CKM with 22 at higher relative abundance in lot L1 and 11 at higher relative abundance in lot A4 (Figure S6). Of those at higher relative abundance at A4, 10 OTUs were shared with those at higher abundance in lot A4 when compared to lot 1l: *Enteractinococcus* (OTU #157), *Arthrobacter* (OTU #69), *Pseudomonas* (OTU #10), *Aeromonas* (OTU #237), *Rhizobium* (OTU #198), *Pectobacterium carotovorum* (OTU #48), *Achromobacter* sp. HJ-31-2 (OTU #16), *Pseudomonas* (OTU #245), *Cloacibacterium* (OTU #72), *Pediococcus* (OTU #50)*.* Additionally*,* of those with higher abundance in 1l two OTUs were shared with those at higher abundance in L1, *Anoxybacillus* (OTU #31) and *Planococcaceae*(OTU #152)*.* Additionally, *Pediococcus* (OTU #50) was at higher relative abundance in A4 for both CKM and CK.

**Table S1**: Descriptions of cigarette products tested at three different experimental conditions (pocket, room, and refrigerator) over time (day 0, 5, 9 and 14).

| **Brands** | **Lot** | **Condition** | **Time Point (Days)** | | | |
| --- | --- | --- | --- | --- | --- | --- |
| NMB | 4C17 | Pocket | D0 (n=2) | D5 (n=2) | D9 (n=2) | D14 (n=2) |
|  |  | Room | D0 (n=2) | D5 (n=2) | D9 (n=2) | D14 (n=2) |
|  |  | Fridge | D0 (n=2) | D5 (n=2) | D9 (n=2) | D14 (n=2) |
|  | 4CO3 | Pocket | D0 (n=2) | D5 (n=2) | D9 (n=2) | D14 (n=2) |
|  |  | Room | D0 (n=2) | D5 (n=2) | D9 (n=2) | D14 (n=2) |
|  |  | Fridge | D0 (n=2) | D5 (n=2) | D9 (n=2) | D14 (n=2) |
|  | 4K01 | Pocket | D0 (n=2) | D5 (n=2) | D9 (n=2) | D14 (n=2) |
|  |  | Room | D0 (n=2) | D5 (n=2) | D9 (n=2) | D14 (n=2) |
|  |  | Fridge | D0 (n=2) | D5 (n=2) | D9 (n=2) | D14 (n=2) |
| CC | A8 | Pocket | D0 (n=2) | D5 (n=2) | D9 (n=2) | D14 (n=2) |
|  |  | Room | D0 (n=2) | D5 (n=2) | D9 (n=2) | D14 (n=2) |
|  |  | Fridge | D0 (n=2) | D5 (n=2) | D9 (n=2) | D14 (n=2) |
|  | B2 | Pocket | D0 (n=2) | D5 (n=2) | D9 (n=2) | D14 (n=2) |
|  |  | Room | D0 (n=2) | D5 (n=2) | D9 (n=2) | D14 (n=2) |
|  |  | Fridge | D0 (n=2) | D5 (n=2) | D9 (n=2) | D14 (n=2) |
|  | B3 | Pocket | D0 (n=2) | D5 (n=2) | D9 (n=2) | D14 (n=2) |
|  |  | Room | D0 (n=2) | D5 (n=2) | D9 (n=2) | D14 (n=2) |
|  |  | Fridge | D0 (n=2) | D5 (n=2) | D9 (n=2) | D14 (n=2) |
| CCM | A8 | Pocket | D0 (n=2) | D5 (n=2) | D9 (n=2) | D14 (n=2) |
|  |  | Room | D0 (n=2) | D5 (n=2) | D9 (n=2) | D14 (n=2) |
|  |  | Fridge | D0 (n=2) | D5 (n=2) | D9 (n=2) | D14 (n=2) |
|  | B2 | Pocket | D0 (n=2) | D5 (n=2) | D9 (n=2) | D14 (n=2) |
|  |  | Room | D0 (n=2) | D5 (n=2) | D9 (n=2) | D14 (n=2) |
|  |  | Fridge | D0 (n=2) | D5 (n=2) | D9 (n=2) | D14 (n=2) |
|  | B3 | Pocket | D0 (n=2) | D5 (n=2) | D9 (n=2) | D14 (n=2) |
|  |  | Room | D0 (n=2) | D5 (n=2) | D9 (n=2) | D14 (n=2) |
|  |  | Fridge | D0 (n=2) | D5 (n=2) | D9 (n=2) | D14 (n=2) |
| CK | A4 | Pocket | D0 (n=2) | D5 (n=2) | D9 (n=2) | D14 (n=2) |
|  |  | Room | D0 (n=2) | D5 (n=2) | D9 (n=2) | D14 (n=2) |
|  |  | Fridge | D0 (n=2) | D5 (n=2) | D9 (n=2) | D14 (n=2) |
|  | Il | Pocket | D0 (n=2) | D5 (n=2) | D9 (n=2) | D14 (n=2) |
|  |  | Room | D0 (n=2) | D5 (n=2) | D9 (n=2) | D14 (n=2) |
|  |  | Fridge | D0 (n=2) | D5 (n=2) | D9 (n=2) | D14 (n=2) |
|  | L1 | Pocket | D0 (n=2) | D5 (n=2) | D9 (n=2) | D14 (n=2) |
|  |  | Room | D0 (n=2) | D5 (n=2) | D9 (n=2) | D14 (n=2) |
|  |  | Fridge | D0 (n=2) | D5 (n=2) | D9 (n=2) | D14 (n=2) |
| CKM | A4 | Pocket | D0 (n=2) | D5 (n=2) | D9 (n=2) | D14 (n=2) |
|  |  | Room | D0 (n=2) | D5 (n=2) | D9 (n=2) | D14 (n=2) |
|  |  | Fridge | D0 (n=2) | D5 (n=2) | D9 (n=2) | D14 (n=2) |
|  | Il | Pocket | D0 (n=2) | D5 (n=2) | D9 (n=2) | D14 (n=2) |
|  |  | Room | D0 (n=2) | D5 (n=2) | D9 (n=2) | D14 (n=2) |
|  |  | Fridge | D0 (n=2) | D5 (n=2) | D9 (n=2) | D14 (n=2) |
|  | L1 | Pocket | D0 (n=2) | D5 (n=2) | D9 (n=2) | D14 (n=2) |
|  |  | Room | D0 (n=2) | D5 (n=2) | D9 (n=2) | D14 (n=2) |
|  |  | Fridge | D0 (n=2) | D5 (n=2) | D9 (n=2) | D14 (n=2) |

**Figure S1**: PCoA analysis plots of Bray-Curtis computed distances between cigarette products. Colored by lot, condition, and time point and tested with ANOSIM. Ellipses are drawn at 95% confidence intervals.

**Figure S2**: PCoA analysis plots of Bray-Curtis computed distances between individual cigarette products CC, CCM, CK and CKM. Colored by (A) condition or (B) lot. Shapes represent time points day 0 (circle), day 5 (square), day 9 (plus sign), and day 14 (triangle). Tested with ANOSIM on individual variables: CC by time point (R=0.06607, *p*=0.002) and lot (R=0.06454, *p*=0.001); CCM by time point (R= 0.08513, *p*=0.001) and lot (R=0.06454, *p*=0.001); NMB by time point (R = .062, *p* = .001), condition (R = .062, *p* =.002), and lot (R = .198, *p* = .001); CK by time point (R = 0.1007, *p* =.002) and lot (R = 0.1762, *p* =.001); and CKM by lot (R = 0.1703, *p* =.001) and time point (R = 0.1865, *p* =.001).

**Figure S3:** Alpha diversity comparison by brand, condition, and time point. Box plots showing Shannon diversity index for mentholated Camel Crush, Camel Crush, mentholated Camel Kings, Camel Kings, and Newport Menthols over time point (Day: D0, D5, D9, D14) and experimental storage condition (pocket, room temperature and refrigerator). The blue line represents a locally estimated scatterplot-smoothed (LOESS) calibration curve with the grey areas representing 95% confidence intervals.

**
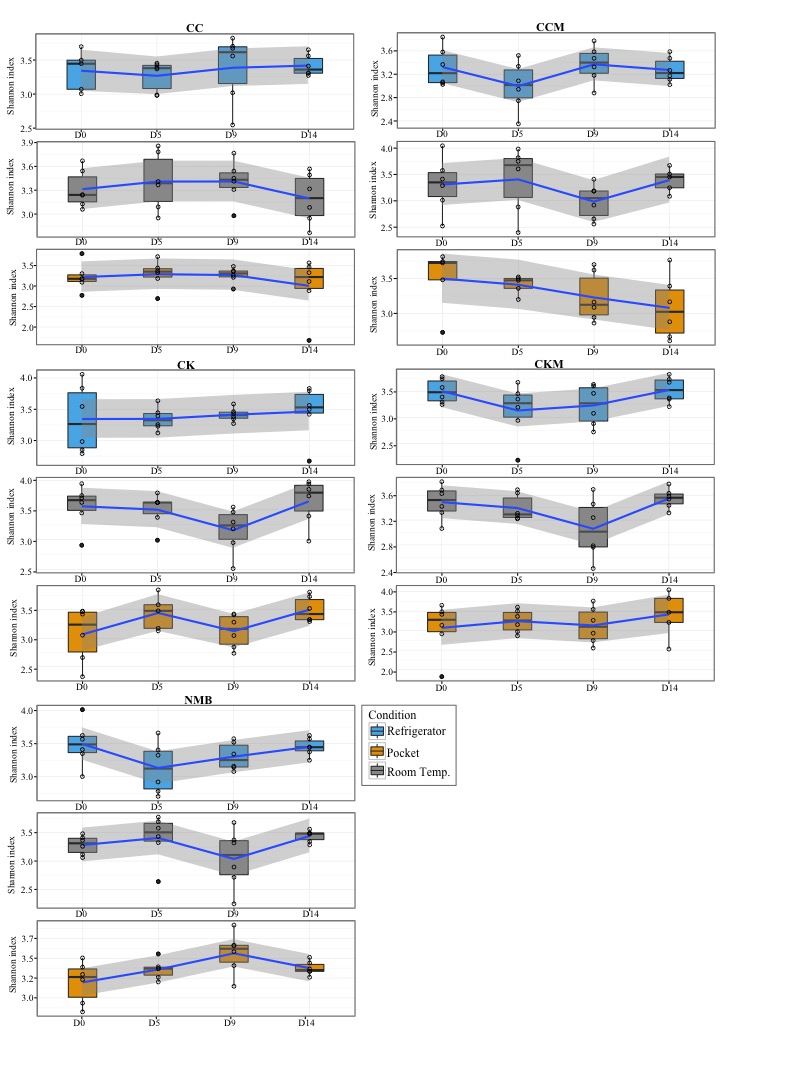
**

**Figure S4:** Overview of relative abundances of bacterial OTUs that were statistically significantly different (p-value < 0.001) between lots (A) AC03 and 4K01 and lots (B) 4C17 and 4K01 for Newport Menthols (NMB). The dotted line highlights the conversion in log2-fold change from negative to positive values.

**Figure S5:** Overview of relative abundances of bacterial OTUs that were statistically significantly different (p-value < 0.001) between lots (A) Il and A4 and lots (B) L1 and A4 for Camel Kings (CK). The dotted line highlights the conversion in log2-fold change from negative to positive values.

**Figure S6:** Overview of relative abundances of bacterial OTUs that were statistically significantly different (p-value < 0.001) between lots (A) Il and A4 and lots (B) L1 and A4 for mentholated Camel Kings (CKM). The dotted line highlights the conversion in log2-fold change from negative to positive values.

**Figure S7**: TSNA levels in μg/g of tobacco over time at refrigerator conditions. Comparison of (A) N-nitrosonornicotine (NNN) and (B) Nicotine-derived nitrosamine ketone (NNK) levels in all brands at day 0 (D0) and day 14 (D14) at pocket conditions. No significant differences (at an alpha level of 0.05) were found between D0 and D14 for a given brand.
